# Acute pancreatitis secondary to spontaneous intramural duodenal hematoma: A case report and a review of the literature^☆^

**DOI:** 10.1016/j.ijscr.2022.107424

**Published:** 2022-07-20

**Authors:** Wissal Skhiri, Marwa Moussaoui, Jamal Saad, Mohamed Maatouk, Asma Chaouch, Ines Mazhoud

**Affiliations:** a Department of Radiology, Fattouma Bourguiba Monastir, Tunisia; b Department of Surgery, CHARLES NICOLE, Tunis, Tunisia

**Keywords:** Acute pancreatitis, Intramural duodenal hematoma, Case report, Review of the literature, IDH, Intramural duodenal hematoma, CT, computed tomography

## Abstract

**Introduction:**

Intramural duodenal hematoma is a rare entity, often secondary to traumatic origin, but more rarely spontaneous due to blood flow disorders, especially in the context of anticoagulant therapy.

**Case presentation:**

We report the case of a 66-year-old woman under anticoagulant treatment for atrial fibrillation, who was diagnosed with acute pancreatitis secondary to a spontaneous duodenal hematoma. The evolution was favorable under medical treatment.

**Discussion:**

Intramural duodenal hematoma is frequently associated with abdominal pain and hematemesis, more rarely, it can be responsible for an acute pancreatitis, which is considerate as an unusual complication. We report here a case of duodenal hematoma revealed by acute pancreatitis along with a review of the literature since 2011.

**Conclusion:**

Monitoring of patients on oral anticoagulants helps prevent the occurrence of IDH and avoid its complications, which can be fatal.

## Highlights

• Spontaneous Intramural duodenal hematoma is a rare entity, especially in the context of anticoagulant therapy
• More rarely, it can be responsible for an unusual complication ' acute pancreatitis'
• Monitoring of patients on oral anticoagulants helps prevent the occurrence of IDH and avoid its complications, which can be fatal

## 1 Introduction

Intramural duodenal hematoma (IDH) is uncommonly noted. It is usually a complication of blunt abdominal trauma, endoscopic biopsy, or peptic ulcer disease; more rarely, it is spontaneous and secondary to blood clotting disorders, especially in the context of anticoagulant therapy [1]. IDH is rarely associated with pancreatic diseases, and the relation between these disorders has not been clarified [2]. We report here a case of duodenal hematoma revealed by acute pancreatitis along with a review of the literature since 2011 [1], [3], [4], [5], [6], [7], [8], [9], [10], [11], [12], [13], [14], [15], [16], [17].

## 2 Case report

**Fig. 1:**
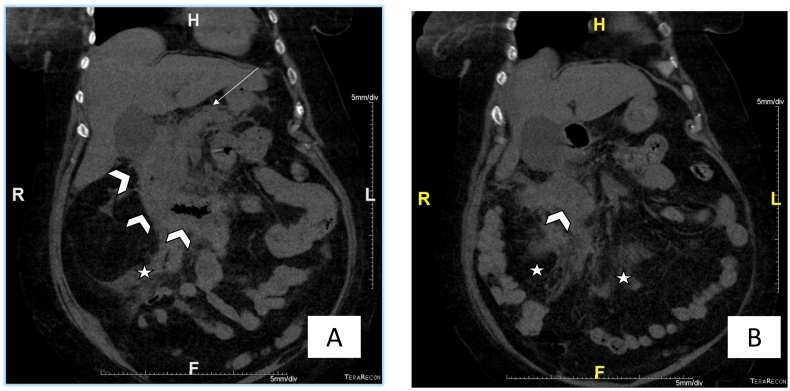
(A,B): CT coronal reconstructions without contrast injection showing a regular circumferential parietal thickening spontaneously hyperdense of the D2, D3 portions of the duodenum (arrowheads), swollen pancreas (arrow) with densification of the mesenteric fat around (star).

**Fig. 2:**
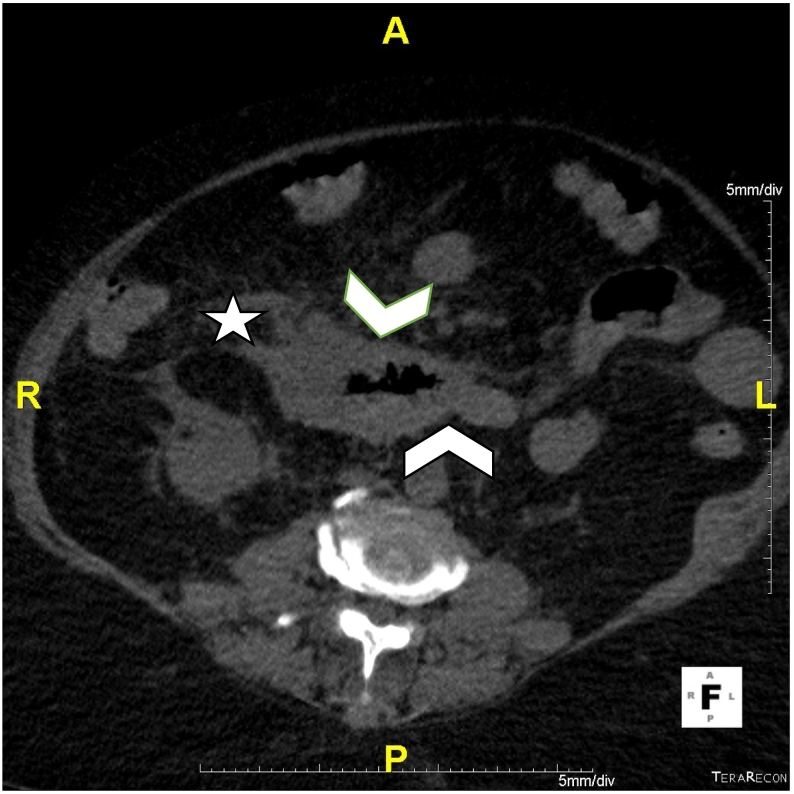
CT axial reconstruction without contrast injection showing a regular circumferential parietal thickening spontaneously hyperdense of the D2, D3 portions of the duodenum (arrowheads) with densification of the surrounding mesenteric fat (star).

**Fig. 3:**
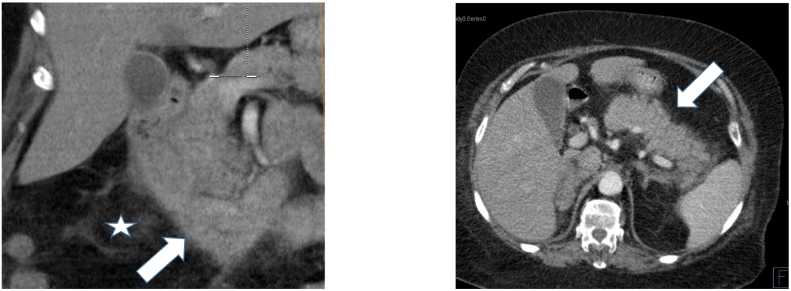
(A): CT coronal reconstruction with contrast injection showing a regular circumferential parietal thickening of the D2, D3 portions of the duodenum (arrowheads), swollen pancreas (arrow) with densification of the mesenteric fat around (star), B: axial reconstruction with contrast injection showing a swollen pancreas (arrow).

**Fig. 4:**
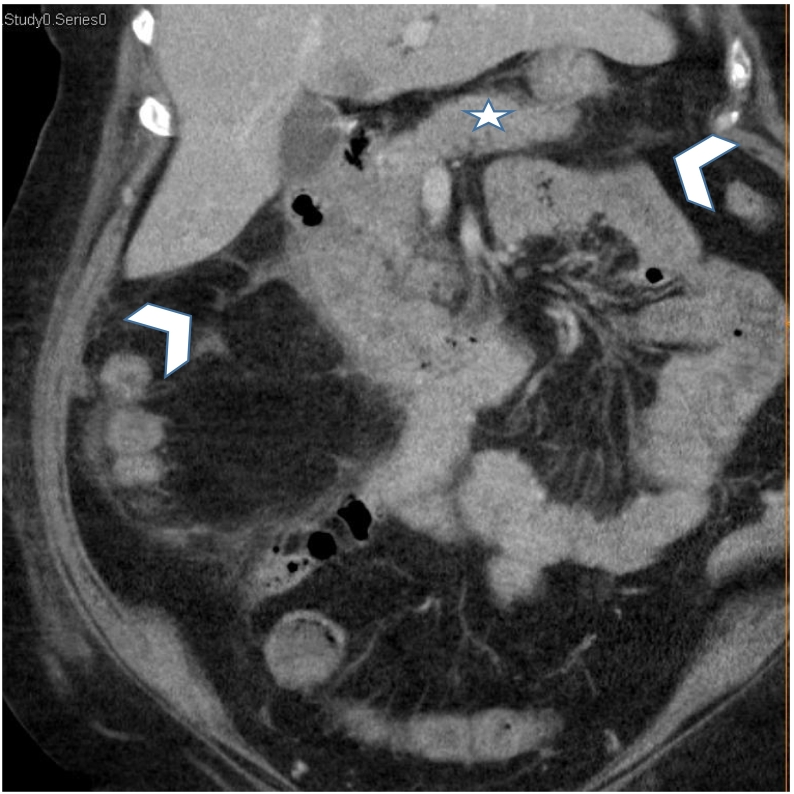
CT coronal reconstructions with contrast injection showing a regression of the regular parietal thickening of the D2, D3 portions of the duodenum and the densification of the mesenteric fat around (arrowheads), the pancreas returns to its normal size (star).

A 66-year-old female with a past medical history of hypertension and atrial fibrillation. She is under anticoagulant therapy, Vitamin K antagonists (VKAs), (Acenocoumarol 4 mg) was admitted to the hospital after experiencing four days of epigastric pain, jaundice and vomiting without any evidence of trauma. The physical examination revealed pale teguments and mucous membranes. Palpation of the epigastric region revealed tenderness. An electrocardiogram was performed to eliminate a myocardial Infarction. Then a pancreatic assessment was requested for the epigastralgia. We suspected also an alteration of the hemostasis balance because of the patient's antecedents. The blood tests showed, anemia with a hemoglobin level of 5.9 g/dl. The prothrombin level was at 12 % and the INR was 3.4 (target INR was between 2 and 3). The ionogram was normal. The lipase blood level was elevated to 500 U/L. The diagnosis of acute pancreatitis was retained. A CT scan (Without and with enhancement) was performed for the classification of acute pancreatitis (Fig. 1, Fig. 2, Fig. 3), it showed a swollen pancreas, with homogeneous enhancement. Densification of peri-pancreatic fat, consistent with acute oedemato-interstitial pancreatitis without peri-pancreatic collection. There was no of dilatation of the intra and extra hepatic bile ducts, nor of the pancreatic duct. The CT scan also showed a circumferential and regular parietal thickening, of D2 and D3, reaching the papilla, spontaneously hyperdense evoking a duodenal hematoma. The IDH extended over 8 cm with 20 mm thickness without significant stenosis. Ultrasound imaging showed no abnormalities in gallbladder nor fatty liver. The diagnosis retained, in view of the clinical, biological and radiological data was a duodenal hematoma complicated by an acute pancreatitis. Therapeutically, the patient was hospitalized in the surgical department, where anticoagulants and parenteral nutrition were stopped along. Hydro-electrolytic rebalancing and blood transfusion were initiated. The evolution was marked by an improvement of the clinical state and of the coagulation disorders. A follow-up abdominal CT scan on day 6 of hospitalization showed regression of the duodenal parietal thickening (Fig. 4). Oral feeding was tolerated at day 20. The work has been reported in line with the SCARE 2020 criteria [18].

## 3 Discussion

IDH was first described in 1838 by Mac Lauchlan. More than 70% of duodenal hematomas are of traumatic origin [1], [3]. IDH can be spontaneous following an overdose of anticoagulant treatment or other circumstances such as endoscopic procedures [4], [5]. The occurrence of IDH due to overdose of anticoagulant treatment is a rare complication, but is increasingly observed due to a wider prescription of anticoagulants in a population with a longer life expectancy [3]. Intramural hematoma affects, in decreasing order of frequency, the jejunum, the ileum, the duodenum (D2 and D3 especially), and more rarely the colon and the esophagus. For the duodenum, the rich submucosal vascular supply and its fixity to the spine explain the susceptibility of the D2 and D3 to be the site of an intramural hematoma [1], [6]. The appearance of abdominal pain and an occlusive and/or hemorrhagic syndrome in a patient receiving antivitamins K, especially if there is an overdose, should prompt consideration of the diagnosis. The association between IDH and pancreatic diseases (acute or chronic pancreatitis, tumor causes) has been described in the literature and it is since 1981 that Van Spreeuwel reported the first case of IDH secondary to an acute attack of chronic pancreatitis. The physiopathology between IDH and pancreatitis is still unclear and several hypotheses have been mentioned [4], [6]: the hematoma may extend to the papilla and cause acute pancreatitis by obstruction, the other hypothesis is vascular where the intra mucosal duodenal vessels may be eroded by the pancreatic enzymes secreted during acute pancreatitis, Therefore, it is difficult to conclude that IDH is due to acute pancreatitis or the opposite, by referring to the radiological data alone and it is only the clinical context and the patient's history that can decide [5], [6]. Shiozawa and al [2], after analyzing all cases of IDH observed with pancreatitis since 1980, re-established a classification associating an IDH and acute pancreatitis. A search was conducted in Pub Med for articles published, in English or French using the following terms: “acute pancreatitis” and “IDH” since 2011, finds 18 cases of IDH associated with acute pancreatitis of which only 8 cases classified “A” according to the Shiozawa classification (Table 1) [4], [5], [7], [8], [9], [10]. The hematoma was spontaneous in one case, secondary to endoscopic hemostasis of a bleeding ulcer in one case, and a complication of anticoagulant therapy in the other 6 cases (i.e. 30 % of cases). Compared with the literature review published by Shiozawa including 33 cases reported between 1980 and 2010, only one observation in which anticoagulant treatment was the cause of IDH (i.e., 3 % of cases). This increase may be explained by the increasing prescription of anticoagulants.

**Table 1:** Published cases of pancreatitis associated with intramural duodenal hematoma.

	Reference	Year	Age/sex	Medical history	Chronic alcoholism	Cause of IHD	Treatment	Type(*)
19	Present case		66F	AF	No	AC	Med	A
18	Figueiredo and al [14]	2019	55 M	None	Yes	AP	Med	B
17	ELGHALI and al [5]	2019	68 M	D, AF	No	AC	Med	A
16	ELGHALI and al [5]	2019	72F	MVR	No	AC	Med	A
15	ELGHALI and al [5]	2019	54F	MVR	No	AC	Med	A
14	OFORI and al [4]	2018	83 M	H, D, AZ	No	Inj	Med	A
13	OLIVIA and al [13]	2017	43 M	AP	Yes	AP	OP	C
12	Eurboonyanun and al [15]	2016	27 M	None	Yes	AP	Med	B
11	Chang and al [6]	2015	43 M	None	Yes	AP	Med	B
10	Elmoghaz and al [11]	2015	31 M	None	Yes	AP-dd	OP	B
9	Gharbi and al [10]	2014	67 M	MVR,H, epilepsy	NO	AC	Med	A
8	Khurana and al [1]	2014	73 F	DVT	No	AC/ADK p/PA	Op	B
7	Veloso and al [9]	2013	64 M	MI	Yes	AC	Med	A
6	Lee CY and al [16]	2013	47 M	D-HVB	No	AP (dengue)	Med	B
5	Dinis Silva and al [8]	2012	80 M	AF/arthrosis	No	Inj	Med	A
4	Goyal and al [7]	2012	29 M	None	Yes	–	Med	A
3	Young Lee and al [12]	2012	55 M	AP	Yes	AP	End	B
2	Fukunaga and al [3]	2011	49 M	None	Yes	AP	OP	B
1	Neuzillet and al [17]	2011	62 M	AP	Yes	AP	OP	C

AP: acute pancreatitis; MVR: mitral valve replacement; AF: atrial fibrillation, MI: myocardial infarction, inj: therapeutic injection for bleeding peptic ulcer, dd: duodenal diverticulum, DVT: deep vein thrombosis, Op: Operation, Endo: Endoscopy, ADK p: pancreatic adenocarcinoma, H: high blood pressure, D: diabetes, AZ: Alzheimer's disease; Med: medical treatment, HVB: viral hepatitis B, AC: anticoagulation.

(*): A classification made by Schiozawa and al, interesting non traumatic IDH associated with pancreatic diseases: A: acute pancreatitis due to duodenal papilla obstruction by hematoma; B: hematoma formation due to vascular disruption by pancreatic enzymes released during acute pancreatitis; and C: hematoma formation due to vascular disruption by pancreatic enzymes released during chronic pancreatitis or its acute exacerbation.

The diagnosis of spontaneous HID complicated by acute pancreatitis is based on a suggestive clinical context and biology which often shows anemia, alteration of the hemostasis balance with elevation of the lipase level [7]. Ultrasound is not very sensitive and sometimes shows a duodenal parietal thickening. IDH presents itself in CT as a circumferential duodenal parietal thickening, more or less stenosing, spontaneously hyperdense measuring between 65 and 95 HU, without enhancement after contrast injection, spontaneus density can be lower in patients under anticoagulant treatment (40 HU). Compression of the biliary and/or pancreatic ducts may cause ductal dilatation. Its main differential diagnosis is duodenal and pancreatic tumors which usually enhance after injection. Liquid level, due to sedimentation of different blood components can be found in IDH. A hyperdense line surrounding the hematoma has been described “ring sign”. On MRI, the comparison of T1 and T2 weighted images allows the recognition of the signal of hemoglobin degradation products according to the age of the lesion. Endoscopy is used to rule out benign or malignant intrinsic stenosis and other causes of bleeding. Therapeutically, medical treatment is sufficient more than two times out of three. It consists in correcting hypocoagulability by stopping anticoagulant treatment, hydrolytic rebalancing and transfusions [3]. The indication for surgery can only be given in the case of a “giant” haematoma with a risk of rupture or in the case of uncontrollable digestive hemorrhage [2], [11]. Minimally invasive procedures such as echo- or scan-guided drainage or endoscopic decompression of the IDH is preferable to laparotomy [4], [7], [12], [13].

## 4 Conclusion

Spontaneous intramural duodenal hematoma is a rare complication of overdosed anticoagulant therapy. It can lead to several complications including acute pancreatitis, gastrointestinal hemorrhage, or cholestasis. We reported a case of a patient with a non-traumatic IDH complicated by acute pancreatitis. We retained this diagnosis on a suggestive clinical context (patient under anticoagulant treatment), on biology (disturbance of the hemostasis balance, hign lipase blood level) and CT imaging. Abdominopelvic CT scan plays an essential role in the diagnosis. Treatment is essentially medical. Monitoring of patients on oral anticoagulants helps prevent the occurrence of IDH and avoid its complications, which can be fatal.

## Source of funding

None.

## Ethical approval

I declare on my honor that the ethical approval has been exempted by my establishment.

## Consent

Written informed consent was obtained from the patient for publication of this case report and accompanying images. A copy of the written consent is available for review by the Editor-in-Chief of this journal on request.

## CRediT authorship contribution statement

Wissal SKHIRI writing original draft

Marwa MOUSSAOUI investigation

Jamal SAAD resources, collecting the data

Mohamed MAATOUK patient follow up

Asma CHAOUCH Visualization

Ines MAZHOUD validation, supervision

## Provenance and peer review

Not commissioned, externally peer-reviewed.

## Declaration of competing interest

None of the authors have any conflict of interest.
